# Genetic Variation of the Nile Soft-Shelled Turtle (*Trionyx triunguis*)

**DOI:** 10.3390/ijms12106418

**Published:** 2011-09-28

**Authors:** Özgür Güçlü, Celal Ulger, Oğuz Türkozan

**Affiliations:** 1 Department of Güzelyurt Vocational School Fermantation, Aksaray University, Güzelyurt/Aksaray 68200, Turkey; E-Mail: ozgguclu@yahoo.com; 2 Department of Biology, Faculty of Science and Arts, Adnan Menderes University, Aydin 09010, Turkey; E-Mail: turkozan@adu.edu.tr

**Keywords:** *Trionyx triunguis*, mtDNA, D-loop, microsatellite, conservation genetics, population genetics

## Abstract

We studied the genetic structure of *Trionyx triunguis* populations from the Mediterranean and African continent based on mtDNA D-loop (776 bp) and nine microsatellite loci. A total of 102 polymorphic sites and 13 mtDNA haplotypes were described. Nucleotide diversity and haplotypes diversity were 0.047 and 0.974 respectively. Both mtDNA and nDNA supported the existence of two main management units as the Mediterranean and Africa. Based on the mtDNA results, the Mediterranean can be divided into two subunits; western Turkey and the eastern Mediterranean.

## 1. Introduction

The Nile soft shell turtle *Trionyx triunguis* mainly lives in fresh or brackish water lakes, rivers and estuaries but there are also records of this interesting animal from the marine habitats [1,2]. Softshell turtles (Trionychidae) are among the most bizarre living turtles, and can be easily distinguished from typical turtles by their lack of scales, flat shells, and highly modified skulls. Some softshell species achieve enormous sizes. For example, East Asian species such as *Rafetus swinhoei* or species of the genus *Chitra* and *Pelochelys* can reach lengths of greater than 1 meter [3]. Most of these large softshells fall into a single monophyletic group [4], and are critically endangered by human activities, primarily by direct harvesting for food [5]. In this way, large softshell turtles are extremely susceptible to rapid decline and are often rare and difficult to study. Consequently, genetic studies of such species generally include only a few samples. However, relative to the other ‘giant’ softshells, the distribution of *T. triunguis* is very broad and in some parts of its range (especially the coasts of Turkey) it can still be found. Like its East Asian counterparts, *T. triunguis* is a species of conservation concern and its populations have been decimated by habitat destruction and harvesting [6–12].

The current distribution of *T. triunguis* includes parts of the Mediterranean and Africa. In the Mediterranean, and perhaps throughout its range, the largest populations are found in Turkey [13–16]. *Trionyx triunguis* mostly lives in estuaries although there are records of the species being found far out in the Mediterranean and Aegean Seas, where it is sometimes caught by fishermen, especially during winter months [1,2,17].

The evolutionary relationship of *T. triunguis* within Trionychidae has been determined through the use of molecular and osteological data [4,18]. Using both nuclear and mitochondrial DNA, Engstrom *et al.* showed that *T. triunguis* is the sister group to a clade of giant trionychids (*Pelochelys + Chitra*), although this inferred relationship was only well-supported in Bayesian analyses, and the species was relatively distantly related to these other giant softshells [5]. Güçlü *et al.* and Gidis *et al.* reported genetic differences between the African and Mediterranean populations of the species, but additional data on the genetic diversity of this species are necessary to define units for conservation and management [19,20].

In the present study, we describe the genetic diversity among Nile soft-shelled turtle populations using nine microsatellite loci previously isolated from *Pelodiscus sinensis* [21] and mtDNA D-loop region. These data are used to assess the population structure of *T. triunguis* in the Africa and the Mediterranean Basin, providing crucial insights into gene flow and conservation units.

## 2. Materials and Methods

### 2.1. Sampling

**Figure 1:**
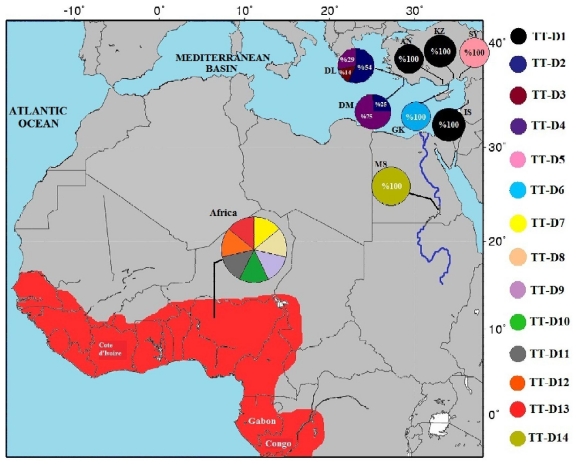
Sampling localities for *T. triunguis* specimens from Mediterranean coasts and African continent and distribution of 14 mtDNA D-loop haplotypes recovered from *Trionyx triunguis* locations (haplotypes labeled TT-D). Pie graphs reflect the frequency of occurrence of each haplotype at each location (Location abbreviations DL-Dalyan, DM-Dalaman, AN-Anamur, GK-Göksu Delta, KZ-Kazanlı, SY-Seyhan, IS-Israel, MS-Egypt).

A total of 102 (52 samples for mtDNA analysis) samples were obtained from 13 different localities in Turkey, Israel, Cote d’Ivoire, Congo and Gabon (Figure 1, Table 1). The Mediterranean samples were collected from Dalyan, Dalaman, (the Western Mediterranean (WT) group); Anamur, Kazanlı, Göksu, Seyhan and Israel, (the East Mediterranean (EM) group), between 2007 and 2009. Samples of unknown localities in Africa were sampled from a captive breeding whereas the Cote d’Ivoire (MNHN 1885–405), Congo (MNHN 1891–361) and Gabon (MNHN 7881) samples the Africa (AG group), were obtained from National d’Histoire Naturelle Muséum in France. Muscle or skin specimens were stored in 95% ethanol.

### 2.2. Mitochondrial DNA Analysis

We assessed the nucleotide variation of relatively fast-evolving fragments of mtDNA control region (D-loop) which is the most variable region of the mitochondrial genome in turtles [22,23]. Total DNA was extracted by a standard phenol/chloroform procedure [24] and a commercial DNA extraction kit (Invitrogen Inc.). Primers were designed based on the mtDNA sequences of *Dogania subplana* (AF366350) and *Pelodiscus sinensis* (AY962573) [25,26]. The mitochondrial D-loop region was amplified via polymerase chain reaction (PCR) using the following primers: OZG (F) 5′-TGG ACT AGT ATA GCA AAG C-3′, OZG (R) 5′-GTC CAG TTT CAT TGA GTT G-3′. PCR amplifications were performed in 50-μL volumes containing 1X KCl PCR buffer (Fermantas Inc.), 1.5 mM MgCl_2_ (Fermantas Inc.), 2.5 mM dNTP, 0.5 mM each primer, 1.0 unit of *Taq* polymerase (Fermantas Inc.), and 1–2 μL (50 ng DNA) of template DNA. PCR conditions were used 95 °C denature for 1 min, 55 °C anneal for 1 min and 72 °C extension for 1 min for 35 cycles for mitochondrial D-loop. Amplicons were purified using the PCR Purification Kit (Invitrogen Inc.) and were analyzed on an AB3700 or 3730xl automatic sequencer using the amplification primers. Sequences were aligned using BioEdit 7.0.9 [27]. Multiple-sequence alignments were done with CLUSTALX [28] using the default parameters.

Five samples were collected from Anamur (An), 1 from Göksu Delta (Gk), 3 from Kazanlı (Kz) and two from the Seyhan River (Sy) and samples from these localities were grouped according to their geographic proximity as AnGkKzSy (11 samples) population. An analysis of molecular variance (AMOVA) [29] was performed to assess the genetic structure and differentiation of *T. triunguis* populations using GenAlEx 6.3 [30]. Statistical significance of the proportion of variance associated with the fixation index, *F_ST_* was determined through permutation tests against a null distribution generated from the data in GenAlEx. The number of migrants (*N_m_*) between each population pair was calculated from genetic distances through the equation *N_m_* = (1 − *F_ST_*)/2*F_ST_* [31].

We examined lineage history in several ways using the D-loop data. The distribution of pairwise differences among individuals, also known as the mismatch distribution, was calculated using ARLEQUIN 3.0 [32]. The shape of mismatch distributions can be used to infer whether a population has undergone sudden population expansion [33,34]. A significant sum of squared differences (SSD; *p* < 0.05) was taken as evidence of population expansion. ARLEQUIN was also used to calculate Tajima’s *D* and Fu’s *Fs* neutrality tests [35,36]. A significant value for *D* may be due to factors such as population expansion, bottlenecks, or heterogeneity of mutation rates [37,38]. A significantly large negative value for *F_S_* may also indicate population expansion [36,39]. The significance of the *D* and *Fs* values was tested by 1000 randomization replicates. Minimum-evolution (ME) analyses were performed using maximum-likelihood pairwise distance by MEGA 5.0 [40].

### 2.3. Microsatellite Analysis

Amplicons were obtained from nine of the 15 microsatellite loci (PS-01, PS-04, PS-11, PS-24, PS-25, PS-28, PS-29, PS-36, PS-40) [21]. One primer for each pair was fluorescently labeled with 6-FAM, HEX or NED. Each locus was amplified using a cycle of 95 °C for 3 min followed by 35 cycles at 94 °C for 1 min, 50–55 °C for 45 s and 72 °C for 30 s, with a final extension of 72 °C for 5 min. Allele length was determined on AB3700 or 3730xl automatic sequencer. Allele sizes were assigned using the Peakscanner package (ABI PRISM Peak scanner^TM^ Software ver. 1.0.).

For each locus, allelic frequencies in between populations and pairwise comparison of the population pairs were conducted using a Markov chain method. Linkage disequilibrium was also tested using the Markov chain permutations and Fisher’s exact test. All of these analyses were carried out using GENEPOP 3.3 [41]. Allelic diversity and observed heterozygosity (*Ho*) and expected heterozygosity (*He*) among populations for each locus were computed using GenAlEx 6.3 [30].

In cases when the sample size was small (*n* < 10) and the number of studied loci was low (*n* < 20), genetic structures of the populations were determined through *F_ST_* [42,43]. Genetic differentiation among populations and groups was calculated using *F_ST_* and pairwise exact tests of genetic differentiation with GenAlEx 6.3 [30]. Measures of gene flow (*Nm*) among and within groups were calculated from pairwise estimates of *F_ST_* (*Nm =* ¼ (1/*F_ST_* − 1)) [44]. Analysis of molecular variance (AMOVA) was used to partition genetic variance among and within groups and populations, based on *F_ST_* calculated in GenAlEx 6.3 [30].

Evidence for recent population bottlenecks was assessed with Bottleneck 1.2 software [45]. In populations that experience population bottlenecks, parallel decreases should be observed in heterozygosity and allele numbers in polymorphic loci. However, this pattern only occurs if loci evolved under the infinite allele model (IAM). If loci evolved under the Stepwise mutation model (SMM), there can be cases when heterozygote excess is not observed [46]. Sign test and Wilcoxon sign-rank tests were applied so as to show whether loci of a population show significant heterozygote excess. These tests were applied using SMM, IAM and TPM models (Two phase model) [45,46]. To examine the genetic relationship among populations, pairwise estimates of Nei’s D were calculated from allele frequencies using TFPGA version 1.3 [47]. These estimates were then subjected to cluster analyses by an unweighted pair group method with arithmetic means (UPGMA).

An assignment test assigns individuals to a population through the genotype formed depending on loci according to Bayesian probability approach [48] implemented in GeneClass2 program [49].

## 3. Results

### 3.1. Mitochondrial DNA Analysis

A total of 776 bp were aligned for 52 individuals. Thirteen mitochondrial DNA haplotypes were found (Figure 1, Gene Bank, Table 1). A total of 102 polymorphic sites were detected consisting of 68 transitions, 12 transversions and 22 insertions/deletions. Samples from Anamur, Kazanlı and Israel, all show a single haplotype (TT-D1) that was not identified in any other locality. Haplotypes TT-D2, TT-D3 and TT-D4 were restricted to WT while all samples from AG had haplotypes that were distinct from the Mediterranean.

Sequence divergences for the mtDNA-D loop were 0.13% between WT and EM, 5.42% between WT and AG, and 5.16% between EM and AG. Nucleotide diversity was 0.047 [50] and haplotype diversity (*H_d_*) was 0.974 for the overall dataset.

The most significant genetic difference between groups was calculated between those in AG and WT, whereas the least significant difference was determined between WT and EM (Genetic distance and *F_ST_* values) (Table 2). Nevertheless, it has been determined that all three regions had significant genetic differences between one another (*p* < 0.001). The AMOVA analysis showed that genetic variation among groups constituted 81% (*p* < 0.01) of the whole variation, whereas the genetic variation among populations was at 19% (*p* < 0.01).

The highest gene flow was between WT and EM while the gene flow between AG and WT was rather limited (Table 3).

Except for the AG, Tajima *D* value was found to be positive in all populations although it was not significant (*p* > 0.05). This shows that populations were not affected by factors such as population growth, selection and bottlenecks in the past. Mismatch analysis rejected the null model of population expansion (SSD; *p* > 0.05).

**Figure 2:**
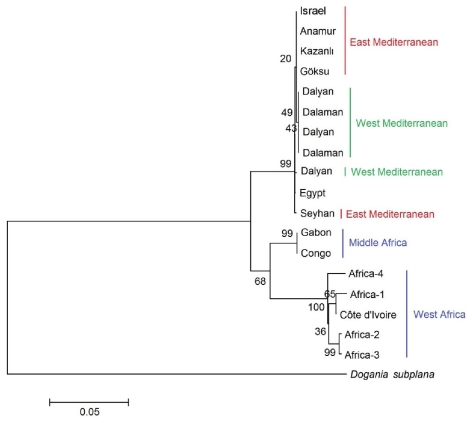
Minimum-evolution (ME) tree for D-loop gene sequence based on maximum likelihood pairwise distance. Numbers indicate bootstrap values of bootstrap replicates (Kimura-2 parameter, 1000 replications).

Based on Nei’s unbiased genetic distance for all haplotype pairs, *T. triunguis* was basically divided into two main clades, namely the Mediterranean basin and the AG. The Mediterranean clade was divided into two subclades; EM, consisting of Israel, Kazanlı, Anamur and Göksu, and WT consisting of Dalyan and Dalaman. The sequence of Amer and Kumazava from Egypt clustered with the Mediterranean clade [51] (Figure 2).

### 3.2. Microsatellite DNA Analysis

We analyzed polymorphism across nine loci in 102 individuals from three populations identifying a total of 15 alleles. The number of alleles per locus ranged from two (PT-04) to four (PT-01) although six microsatellites were fixed for one allele. Observed heterozygosity did not show a statistical difference from expected heterozygosity under the Hardy Weinberg equation. Observed heterozygosity range was 0.12 and 0.05, whereas expected heterozygosity was between 0.20 and 0.06. Mean values of *He* and *Ho* were not different among populations (*p* > 0.05). Linkage disequilibrium was not determined between two loci of any single population (*p* > 0.05). Nevertheless, allelic diversity in microsatellite loci occurred non-independently from each other. Significant differences were determined in terms of allele frequencies (*p* < 0.001) between populations.

According to *F_ST_* estimations, significant genetic differences were determined between groups and populations (Table 2). Forty-six percent of the variation was among groups (*p* < 0.01), 3% was among populations within groups (*p* > 0.05), and the remaining 51% was within the populations (*p* < 0.01). There was no measurable genetic differences between AnGkKzSy population and Dalyan and Dalaman populations (Dalyan-AnGkKzSy, *F_ST_* = 0.013, *p* = 0.20; Dalaman-AnGkKzSy, *F_ST_* = 0.021, *p* = 0.12), whereas a significant difference was determined between AnGkKzSy and Israel populations (*F_ST_* = 0.136, *p* < 0.05).

**Figure 3:**
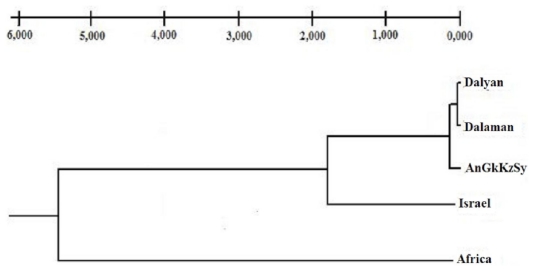
Results of unweighted pair group method with arithmetic means **(**UPGMA) analysis for *T. triunguis* populations based unbiased distance calculated from nine microsatellite loci.

A UPGMA tree generated based on the genetic distance data between populations (Figure 3) displayed two main clades, namely Africa and Mediterranean Basin. Unlike mtDNA, the AnGkKzSy population clustered with the WT along with Dalyan and Dalaman populations.

Gene flow between AG and those from WT and EM is rather limited (*Nm* < 1). On the other hand, there is more gene flow between WT and EM (Table 3). Depending on the *F_ST_* values, gene flow between AnGkKzSy and Dalyan and Dalaman populations is high (Dalyan/AnGkKzSy, *Nm* = 18.4; Dalaman/AnGkKzSy, *Nm* = 11.8). As a result of the Sing and Wilcoxon sign-rank tests performed for all groups and populations, no significant heterozygosity excess was determined in loci (*p* > 0.05). The values calculated under IAM, SMM and TPM models showed that the studied populations of *T. triunguis* did not suffer a bottleneck in the past. According to assignment test, all individuals in each group were classified correctly (Table 4).

## 4. Discussion

### 4.1. Genetic Diversity and Genetic Structure

In contrast to our results, Gidiş *et al.* found no variation in terms of *cyt b* sequences among Mediterranean and African softshell turtles [20]. Furthermore, they could not provide amplicon from Cameroon specimens with their D-loop primers. However, they found low levels of genetic divergence across five nuclear gene sequences. According to our results, based on mtDNA D-loop analysis of current data, the identified haplotypes are unique to the groups; however, genetic differences between groups are essential (*p* < 0.01). In addition, AG has higher genetic diversity compared to WT and EM based on the number of observed haplotypes and alleles.

Despite the fact that we analyzed nine loci, only three were useful for population level analysis. The number of these loci is limited for population genetic analysis. The most important feature in determining the genetic diversity of a species or a population is the amount of heterozygosity in that species or population [52]. The closeness of expected and observed heterozygosity in *T. triunguis* populations shows that these populations are at Hardy-Weinberg equilibrium. According to both mtDNA and microsatellite locus analyses, there is significant genetic difference among the three groups of *T. triunguis* (*p* < 0.01); nevertheless, the AG was completely isolated from WT and EM. Both mtDNA and microsatellite analyses proved a significant genetic difference among 3 groups (*p* < 0.01) and AG seems to be isolated from WT and EM. Lower genetic diversity observed in WT and EM compared to those in AG mean that these populations have small population sizes and probably a decrease in diversity has occurred through founder effect. Genetic diversity in populations and, as a result, allelic richness, has a positive relationship with population size [53]. The AG, therefore, may turn into the one with the largest population size since it has the highest genetic diversity.

According to mtDNA data, although gene flow between WT and EM is seems to be rather limited (*Nm* = 0.3), there is a gene flow between these groups for microsatellite loci (*Nm* = 1.8). Such high gene flow observed for microsatellite loci is a function of the gene flow between AnGkKzSy and Dalyan and Dalaman populations (AnGkKzSy/Dalyan-*Nm* = 18.4; AnGkKzSy/Dalaman-*Nm* = 11.8). The limited gene flow in terms of mtDNA possibly resulted from nesting site fidelity of the females as in the case of marine turtles [54] or due to faster fixation rate of mtDNA. The higher gene flow in terms of microsatellites shows the existence of male mediated gene flow between those populations.

The facts that (i) gene flow estimates based on both mtDNA and microsatellite loci is negligibly low between AG and other populations, (ii) there are haplotypes and alleles unique to this population, and (iii) there are fixed differences between groups showing that the AG occupied the Mediterranean Basin in the past of their evolutionary history. This differentiation determined for *T. triunguis* is also supported by mtDNA *ND4* and *cytochrome b* gene regions [19].

Gidiş *et al.* indicated that there could be gene flow between the Nile River and Middle African populations because of the connection between the Nile and Congo rivers [20]. But, according to D-loop sequences, African samples (including Congo and Gabon) are likely to isolate from the Nile River and Mediterranean Basin. This situation can be clarified with more samples from Africa continent.

### 4.2. Management Strategies

Classification of a population as Evolutionarily Significant Units (ESU) or Management Units (MU) can be possible by determination of the genetic structure of that population [55,56]. Local genetic configurations can be evaluated as important determinant factors for conservation planning and management of a species. In a population divided into sub-populations, any decrease in population size or habitat components cause significant decrease in total number of individuals as well as local disappearances depending on the increase in loss of genetic diversity [57]. Gidiş *et al.* suggested that there are no significant differences between the Mediterranean and African populations and therefore they need to consider combining them into a single conservation unit [20]. However, in our study, both mtDNA and nDNA supported the existence of two main MUs, as AG and the Mediterranean basin for the conservation of *T. triunguis*. Furthermore, based on mtDNA the Mediterranean basin also can be divided in two subMUs (WT and EM). The AG has the highest genetic diversity among all and constitutes the foundation for the evolutionary future of *T. triunguis*.

In recent years the populations of *Trionyx triunguis* has been declining due to habitat degradation and fragmentation. For instance, until the early 1990s, *T. triunguis* had a robust population in terms of number of individuals in Nahal Alexander, a population of Israel from the EM. However, after the sea infiltrated the land due to storms, habitat changes occurred and the population began to decrease rapidly [58]. Such habitat degradations or fragmentations increase the risk of genetic drift and inbreeding in the populations. In such cases, simply restoring habitats is not enough for the populations to recover. For the conservation and management of the species, a multidisciplinary approach is needed and genetic work is one aspect of this information gap.

## 5. Conclusions

We defined two main MUs for *Trionyx triunguis* and provided inventory genetic data for future population restoration for the recovery of this declining species.

## Figures and Tables

**Table 1:** Number of each mtDNA haplotype present in three population samples of Nile soft-shelled turtle (*n* = sample size).

	West Turkey (WT)		East Mediterranean (EM)		African Continent (AG)	Gene Bank Accesion Number
	
	Dalyan (*n* = 7)	Dalaman (*n* = 20)	AnGkKzSy (*n* = 11)	Israel (*n* = 8)	(*n* = 7)	
TT-D1			8	8		HM068069
TT-D2	4	5				HM068070
TT-D3	1					HM068071
TT-D4	2	15				HM068072
TT-D5			2			HM068073
TT-D6			1			HM068074
TT-D7					1	HM068075
TT-D8					1	HM068076
TT-D9					1	HM068077
TT-D10					1	HM068078
TT-D11					1	HM068079
TT-D12					1	HM068080
TT-D13					1	HM068081

**Table 2:** Pairwise comparison of populations (fixation index (*F_ST_*) values based on the analysis of three populations).

	WT	EM	AG
	
**WT**		0.123 *	0.571 *
**EM**	0.648 *		0.375 *
**AG**	0.878 *	0.848 *	

The cells above the shaded areas show genetic distances based on microsatellite (*F_ST_* values), and those below the shaded areas show genetic distances based on mtDNA (*F_ST_* values).

* *p* < 0.001.

**Table 3:** Estimates of gene flow among populations based both mtDNA and nDNA.

	WT	EM	AG
**WT**		1.784	0.188
**EM**	0.27		0.417
**AG**	0.07	0.09	

Cells above the shaded areas show gene flow based on microsatellite (*F_ST_* values), and those below the shaded areas show gene flow based on mtDNA (*F_ST_* values).

**Table 4:** Number of samples assigned to their population of origin based on multilocus genotype (WT-West Turkey Coast, EM-East Mediterranean Coast, AG-African Group).

	Dalyan	Dalaman	WT	AnGkKzSy	Israel	EM	AG	Proportion correctly assigned (%)
	
	*n* = 57	*n* = 20	*n* = 77	*n* = 11	*n* = 7	*n* = 18	*n* = 7	
**Dalyan**	52	5						92.23
**Dalaman**	11	9						45.00
**WT**			77					100.00
**AnGkKzSy**	2	1		8				72.72
**Israel**					7			100.00
**EM**						18		100.00
**AG**							7	100.00

**Mean**								**82.0**
